# Longitudinal MRI contrast enhanced monitoring of early tumour development with manganese chloride (MnCl_2_) and superparamagnetic iron oxide nanoparticles (SPIOs) in a CT1258 based *in vivo* model of prostate cancer

**DOI:** 10.1186/1471-2407-12-284

**Published:** 2012-07-11

**Authors:** Katharina A Sterenczak, Martin Meier, Silke Glage, Matthias Meyer, Saskia Willenbrock, Patrick Wefstaedt, Martina Dorsch, Jörn Bullerdiek, Hugo Murua Escobar, Hans Hedrich, Ingo Nolte

**Affiliations:** 1 Small Animal Clinic and Research Cluster of Excellence “REBIRTH”, University of Veterinary Medicine Hannover, Hannover, Germany; 2 Center for Human Genetics, University of Bremen, Bremen, Germany; 3 Institute of Laboratory Animal Science and Research Cluster of Excellence “REBIRTH” (AG36), Hannover Medical School, Hannover, Germany; 4 Institute of Laboratory Animal Science, Hannover Medical School, Hannover, Germany

## Abstract

**Background:**

Cell lines represent a key tool in cancer research allowing the generation of neoplasias which resemble initial tumours in *in-vivo* animal models. The characterisation of early tumour development is of major interest in order to evaluate the efficacy of therapeutic agents. Magnetic resonance imaging (MRI) based *in-vivo* characterisation allows visualisation and characterisation of tumour development in early stages prior to manual palpation. Contrast agents for MRI such as superparamagnetic iron oxide nanoparticles (SPIOs) and manganese chloride (MnCl_2_) represent powerful tools for the *in-vivo* characterisation of early stage tumours. In this experimental study, we labelled prostate cancer cells with MnCl_2_ or SPIOs *in vitro* and used 1 T MRI for tracing labelled cells *in-vitro* and 7 T MRI for tracking in an *in-vivo* animal model.

**Methods:**

Labelling of prostate cancer cells CT1258 was established *in-vitro* with MnCl_2_ and SPIOs. *In-vitro* detection of labelled cells in an agar phantom was carried out through 1 T MRI while *in-vivo* detection was performed using 7 T MRI after subcutaneous (s.c.) injection of labelled cells into NOD-Scid mice (n = 20). The animals were scanned in regular intervals until euthanization. The respective tumour volumes were analysed and corresponding tumour masses were subjected to histologic examination.

**Results:**

MnCl_2_*in-vitro* labelling resulted in no significant metabolic effects on proliferation and cell vitality. *In-vitro* detection-limit accounted 10^5^ cells for MnCl_2_ as well as for SPIOs labelling. *In-vivo* 7 T MRI scans allowed detection of 10^3^ and 10^4^ cells. *In-vivo* MnCl_2_ labelled cells were detectable from days 4–16 while SPIO labelling allowed detection until 4 days after s.c. injection. MnCl_2_ labelled cells were highly tumourigenic in NOD-Scid mice and the tumour volume development was characterised in a time dependent manner. The amount of injected cells correlated with tumour size development and disease progression. Histological analysis of the induced tumour masses demonstrated characteristic morphologies of prostate adenocarcinoma.

**Conclusions:**

To the best of our knowledge, this is the first study reporting direct *in-vitro* MnCl_2_ labelling and 7 T based *in-vivo* MRI tracing of cancer cells in a model of prostate cancer. MnCl_2_ labelling was found to be suitable for *in-vivo* tracing allowing long detection periods. The labelled cells kept their highly tumourigenic potential *in-vivo.* Tumour volume development was visualised prior to manual palpation allowing tumour characterisation in early stages of the disease.

## Background

Prostate cancer is the second most common form of cancer and the sixth leading cause of cancer deaths among males worldwide [1,2]. During the last decade, several human prostate cancer cell lines were established and of these, DU 145, LNCaP and PC-3 represent the most prevalent lines [3-5]. In contrast to humans, in animals occurrence of prostate cancer is uncommon. In larger non-human mammalians, only the dog is known to develop prostatic cancer with considerable numbers. Canine prostate cancer shares many characteristics with its human counterpart regarding its clinical presentation and pathogenesis. These characteristics include high grade prostatic intraepithelial neoplasia (PIN) which represents the most common precursor of human prostate cancers [6-8], as well as the metastatic pattern and increased incidence with age [6,9,10]. Nevertheless, unlike the human form, canine prostate cancer is an uncommon neoplasm [7] which does not appear to respond to androgen deprivation [11], and most canine prostate cancers do not express the androgen receptor [12]. The prognosis is poor due to late diagnosis and treatment options remain palliative in most cases. Nevertheless, the dog displays a unique model of prostate cancer and the characterisation of therapeutic approaches could be of benefit for human as well as for veterinarian patients.

Currently, five canine prostate cancer cell lines have been published including CPA 1, CT1258, DPC-I, Ace-1, and Leo [13-18]. Until now, the histological type, the karyotype and the *in vivo* behaviour of the CT1258 cell line, which was shown to be highly tumourigenic, were characterised [13,14]. Thereby, the induced tumours mimicked histopathologic and cytogenetic characteristics of the original tumour [14].

In terms of experimental comparability, this study aimed at the controlled delivery of CT1258 cells and early *in vivo* characterisation of tumour development without the need of sacrificing animals. The *in vivo* localisation, migrative behaviour and progression of the tumour growth after injection of CT1258 cells were monitored in regular time periods via 7 T MRI. Thereby, the cells were labelled *in vitro* with the MRI contrast agents manganese chloride (MnCl_2_) and superparamagnetic iron oxide nanoparticles (SPIOs).

Iron oxide nanoparticles are widely used as MRI contrast agents. Scientific and clinical applications include the tracking of labelled transplanted stem cells in neurological and cardiovascular diseases as well as diagnostic imaging of liver and spleen for tumour detection and staging [19-23]. In prostate cancer, labelling of the human cell line PC-3 with lipid-coated SPIOs has been reported [24] and *in vitro* labelling with micron sized iron oxide nanoparticles (MPIOs) and subsequent *in vivo* tracing via MRI was performed in the rodent prostate cancer cell line TRAMPC1 [25].

In contrast, the use of MnCl_2_ for cell labelling and non-invasive *in vivo* cell tracing is much less common. In most cases, manganese-enhanced MRI (MEMRI) has been used in studies of the anatomy and function of the central nervous system and the heart after systemic administration of manganese [26-29]. So far, *in vitro* labelling of cells with MnCl_2_ for MR imaging was performed in murine pancreatic beta cells, human lymphocytes, embryonic stem cells and bone marrow stromal cells [23,30-33]. The manganese agent Mn (III)-transferrin was used for labelling and *in vivo* detection of murine hepatocytes [34]. The use of manganese oxide (MnO) for *in vitro* cell labelling was evaluated in human cell lines including a prostate adenocarcinoma cell line. Labelling with MnO and subsequent MRI-based *in vivo* tracking has been carried out in rat glioma cells [35,36].

The aim of the present study was to evaluate appropriate labelling parameters which enable MR imaging of cells with MnCl_2_ and SPIOs, in a CT1258-based *in vivo* model of canine prostate cancer. This is the first study, reporting direct *in vitro* cell labelling with MnCl_2_ and subsequent MRI-based *in vivo* tracing of prostate cancer cells. This technique enables a time-dependent characterisation of tumour development in regard to tumour size prior to manual palpation in early stages of the disease.

## Results

### Cell viability and proliferation assays

Cell viability was analysed 24 h after the labelling reactions. Labelling for 1.5 h with 0.02 M to 0.04 M MnCl_2_ revealed the percentages of living cells ranging from 91.7% to 23% (tab. 1). The unlabelled control cells revealed 95% of living cells (Table 1). Cells labelled with 0.035 M MnCl_2_ for different time periods (1.5 h to over night) revealed viability values from 89% to 37% (Table 2). Respective control cells revealed 98% of living cells (tab 2).

**Table 1:** **Trypane-blue based cell viability test after labelling of CT1258 cells with different concentrations of MnCl**_**2**_**for 1.5 h**

**Concentration of MnCl**_**2**_**[M]**	**Cell viability [%]**
0 (control)	95
0.02	91.7
0.025	83
0.03	91
0.035	90
0.04	32
0.045	23

**Table 2:** **Cell viability test after labelling of CT1258 cells with 0.035 M MnCl**_**2**_**for different incubation times**

**Incubation time [h]**	**Cell viability [%]**
0 (control)	98
1.5	89
3	86
4.5	81
6	80
Over night	37

CT1258 cells were labelled with 0.035 M MnCl_2_ for different incubation times at 37°C and 5% CO_2_. After labelling, the cells were further cultivated overnight in culture medium at 37°C and 5% CO_2_. Cell viability test was performed with Trypane-blue dye.

Cell proliferation levels that were measured after labelling with 0.035 M MnCl_2_ for 1.5 h revealed mean values of 0.09413 for labelled cells and 0.1128 for control cells (mean values were calculated from 16 measurements per group). Statistical *t* test analysis resulted in a p value of p = 0,008 showing statistical significant difference between both groups.

### *In vitro* 1 T MRI scans

**Figure 1:**
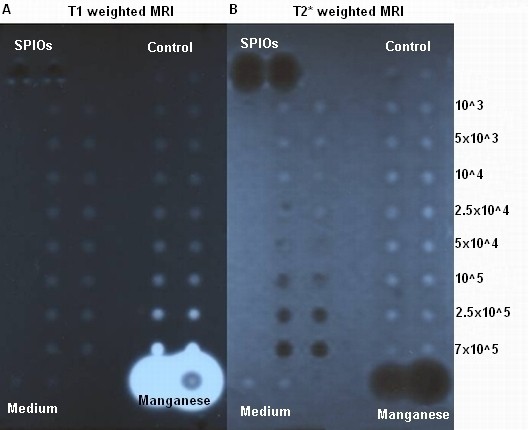
**1T MRI scan of MnCl**_**2**_**and SPIOs labelled cells in a 1% agar phantom.** The numbers of labelled cells accounted: 7 × 10^5^, 2.5 × 10^5^, 10^5^, 5 × 10^4^, 2.5 × 10^4^, 10^4^, 5 × 10^3^, 10^3^. As controls 1 × 10^5^ unlabelled cells, the culture medium, 1 M MnCl_2_, and Endorem solution were used. **A**: T1 weighted MRI. SPIOs labelled cells (left two lanes), the controls with un-labelled cells and the culture medium showed comparable signals. 1 M MnCl_2_ solution showed a strong signal enhancement. MnCl_2_ labelled cells (right two lanes) were detected to a limit of 10^5^ cells. **B**: T2* weighted MRI scan. MnCl_2_ labelled cells (right two lanes), the controls, and the culture medium showed comparable signals. Endorem solution showed a strong signal extinction. SPIOs labelled cells (left two lanes) were detected to a limit of 10^5^cells.

*In vitro* MRI (1 T) examination of the agar block revealed identical detection limits of 10^5^ cells/well for cells labelled with MnCl_2_ (T1-weighted sequences; Figure 1 A) and SPIOs (T2/T2*-weighted sequences; Figure 1 B).

### *In vivo* inoculations, *in vivo* 7 T MRI scans and analysis of tumour volumes

Based on the previous described *in vitro* results, we inoculated two NOD-Scid mice with 10^4^ unlabelled cells subcutaneously prior to the MRI contrast *in vivo* studies. The induced tumours were detectable via manual palpation on day 28 and the animals were sacrificed on day 32 and 40 due to tumour burden. The observed tumour diameters at the scarification date were 10 mm in the respective animals.

**Figure 2:**
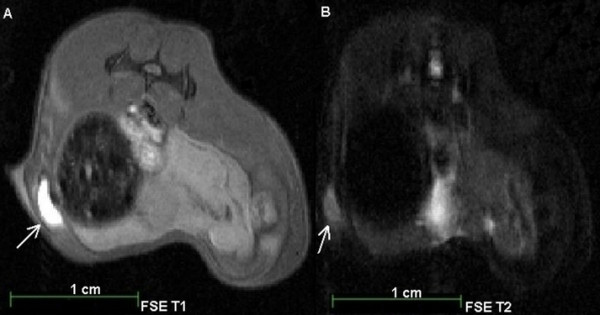
**7T MRI of a male NOD-scid mouse after subcutaneous injection of 10**^**4**^**MnCl**_**2**_**labelled CT1258 cells.** The MRI scans were performed on the same day as the injection (day 1). A: FSE T1 weighted MRI Scan. B: FSE T2 weighted MRI scan. Arrows: localisation of the injected MnCl_2_ labelled cells in T1 (A) and T2 (B) weighted MRI. In T1 weighted MRI (A) the MnCl_2_ labelled cells were detected due to a strong signal enhancement.

**Figure 3:**
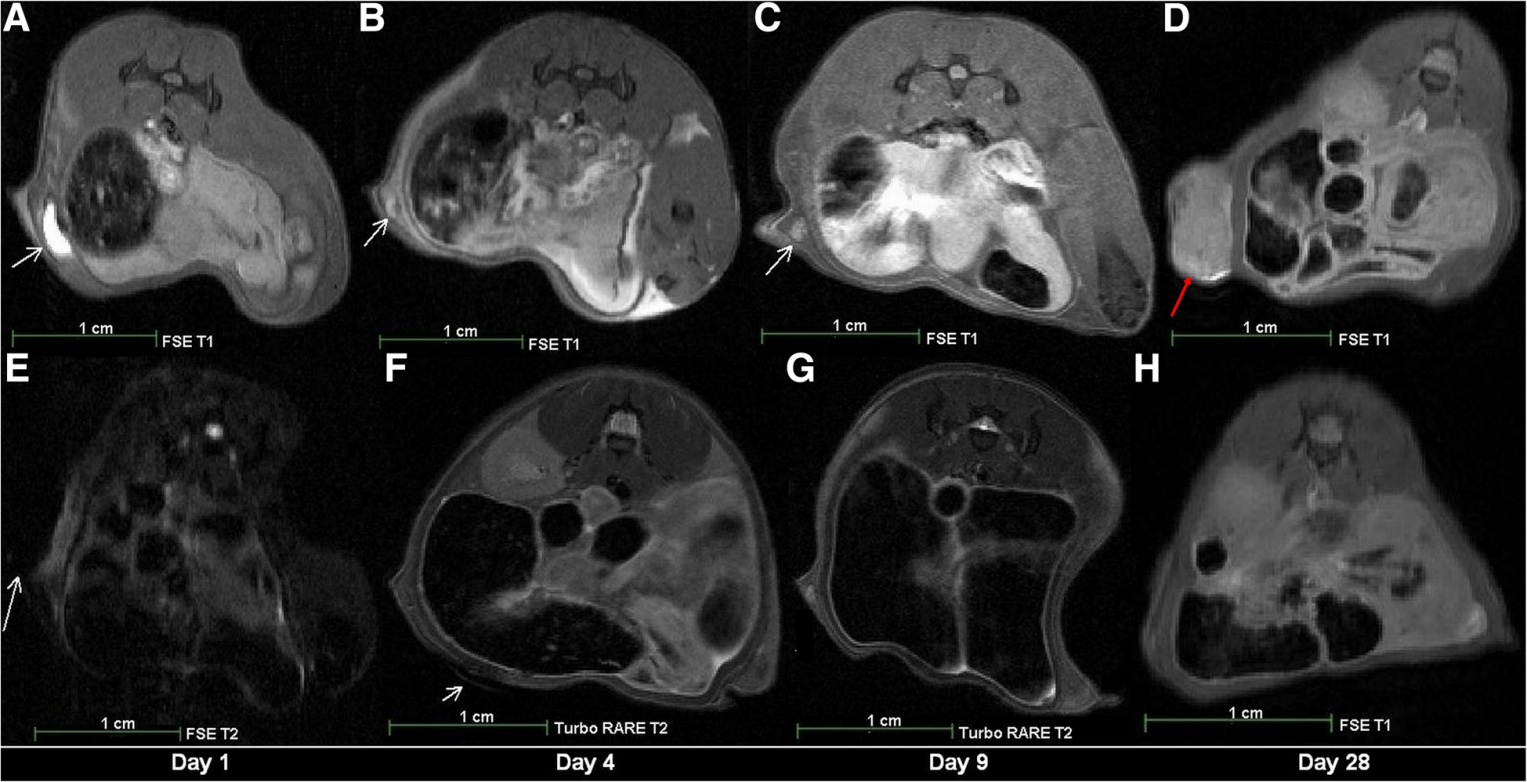
**7T MRI scans of a male NOD-scid mouse after subcutaneous injection of 10**^**4**^**SPIOs labelled CT1258 cells.** The MRI scans were performed on the same day as the injection (day 1). **A**: FSE T1 weighted MRI Scan. **B**: FSE T2 weighted MRI scan. **C**: FISP T2* weighted MRI scan. Arrows: localisation of the injected SPIOs labelled cells in T1 (A), T2 (B) and T2* (C) weighted MRI. In T2 (B) and T2* (C) weighted MRI the SPIOs labelled cells were detected due to a strong signal extinguishment.

In the first MRI group (n = 2) of animals, MnCl_2_ and SPIOs labelled cells were detected *in vivo*. MnCl_2_ labelled cells were detected through signal enhancements in T1 weighted MRI on day 1 (Figures 2 A, B; Figure 3 A), day 4 (Figure 3 B) and day 9 (Figure 3 C) after contrast-agent injection. On day 28, a tumour mass was identified in the animal (Figure 3 D).

**Figure 4:**
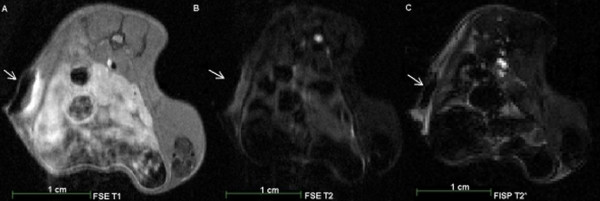
**Longitudinal*in vivo*7 T MRI scans of two male NOD-scid mice after subcutaneous injection of 10**^**4**^**CT1258 cells labelled with either MnCl**_**2**_**or SPIOs at the day 1, 4, 9 and 28 after injection.A-D**: *in vivo* MRI scans of an animal which received MnCl_2_ labelled cells. **A**: FSE T1 weighted MRI scan on the day of injection (day 1). **B**: FSE T1 weighted MRI scan on day 4 after injection. **C**: FSE T1 weighted MRI scan on day 9 after injection. **D**: FSE T1 weighted MRI scan on day 28 after injection. The MnCl_2_ labelled cells were not detectable and a tumour mass developed at the site of injection. **E-H**: *in vivo* MRI scans of an animal which received SPIOs labelled cells. **E**: FSE T2 weighted MRI scan on the day of injection (day 1). **F**: Turbo RARE T2 weighted MRI scan on day 4 after injection. **G**: Turbo RARE T2 weighted MRI scan on day 9 after injection. The cells were not detected and there were no signs of tumour development. **H**: FSE T1 weighted MRI scan on day 28 after injection. White arrows: localisation of labelled cells. Red arrow: localisation of the developed tumour at the site of injection.

**Figure 5:**
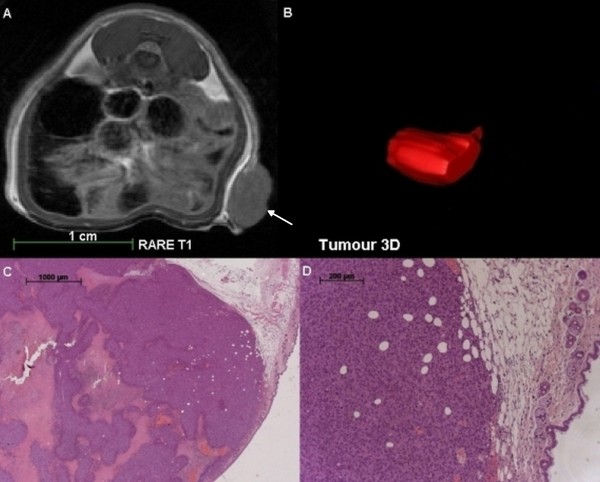
**T1 weighted MRI scan, three dimensional reconstruction and histological analyses of a tumour induced by injection of MnCl**_**2**_**labelled CT1258 cells into a male NOD-scid mouse.A**: RARE T1 weighted *in vivo* MRI scan 24 days after sc injection of the labelled cells. On the right abdominal flank, a tumour mass developed. No signs of metastasis were detected. **B**: Three dimensional graphical analysis of the tumour structure 24 days after injection of the labelled cells. **C** and **D**: Histological analysis of the obtained tumour mass. The obtained tumour showed the characteristic appearance and morphology of a prostate adenocarcinoma. The analysis displayed pleomorphic cells with a high mitotic index. The centre of the obtained tumour was highly necrotic and the tumour infiltrated into the subcutaneous fat tissue. The tumour developed an incomplete fibrous capsule. White arrow: localisation of the developed tumour at the site of injection.

SPIOs labelled cells were detected through signal extinctions in T2/T2* weighted MRI on day 1 (Figures 4 A, B, C; Figure 3 E), and day 4 (Figure 3 F) after contrast-agent injection. On day 9, no signal extinctions were identified and the animal did not show evidence of tumour development (Figure 3 G, H). Within the second group of animals (n = 18), in all animals of the first subgroup (n = 8) MnCl_2_ labelled cells were detected in T1-weighted MRI on the first day after injection. Due to tumour burden the number of animals available on days 4 and 8 decreased and on day 10 two remaining animals showed slight signal enhancements indicating the location of the injected cells. Tumour development occurred in all animals and these tumours were detected prior to manual palpation through T1 and T2 weighted MRI. In nine animals of the second subgroup (n = 10), MnCl_2_ labelled cells were detected on the second day after injection. On days 10 and 16, the number of animals decreased to remaining two animals on day 16. In these animals, slight signal enhancements were detected indicating the presence of MnCl_2_ labelled cells. In five from ten animals, tumours developed which were detected in T1 and T2 weighted MRI prior to manual palpation. Figure 5 shows a T1 weighted MRI scan of a male animal that developed a tumour at the site of injection 24 days after injection (Figure 5 A).

Altogether, in 13 of 18 animals, tumour masses developed at the site of injection. All male animals which had received 10^4^ cells (n = 4) developed tumours (100%) while injection of 10^3^ cells (6 female, 8 male) led to tumour development in 4 female (66%) and 5 male (62.5%) animals. The increment of tumour volumes in [mm^3^/day] was analysed for each animal and is shown in Tables 3, 4, and 5. The animals were numbered from 1 to 13 and grouped according to their gender and amount of injected cells. Table 3 shows increment of tumour growth in animals 1–4 (4 male; 2 intact, 2 castrated; 10^4^ cells). Table 4 displays increment of tumour growth in animals 5–9 (5 male, 3 intact, 2 castrated, 10^3^ cells). Table 5 presents the increment of tumour volumes in animals 10–13 (4 female, 2 intact, 2 ovariohysterectomised, 10^3^ cells).

**Table 3:** **Analysis of tumour volumes detected via 7 T** ***in vivo*** **MRI in a CT1258 based** ***in vivo*** **model of prostate cancer**

**Animal**	**Day 17;**	**Day 22;**	**Day 24;**
	**Tumour volume [mm**^**3**^**]**	**Tumour volume [mm**^**3**^**]**	**Tumour volume [mm**^**3**^**]**
**1**	5.22	124.02	276.13
(♂, intact)			
**2**	5.26	71.90	148.58
(♂, intact)			
**3**	1.61	40.59	66.14
(♂, castrated)			
**4**	2.12	120.51	39.74
(♂, castrated)	1.03	32.70	69.87

The male animals received 10^4^ MnCl_2_ labelled cells. The tumour volumes are displayed for the respective days of MRI measurements after subcutaneous injection. In animal 4, tumour masses developed on the left and right abdominal flanks due to incorrect injection of cells.

♂ = male animal.

**Table 4:** **Analysis of tumour volumes detected via 7 T** ***in vivo*** **MRI in a CT1258 based** ***in vivo*** **model of prostate cancer**

**Animal**	**Day 17;**	**Day 22;**	**Day 24;**	**Day 25;**	**Day 39;**	**Day 43;**	**Day 53;**	**Day 59;**	**Day 66;**
	**Tumour volume [mm**^**3**^**]**	**Tumour volume [mm**^**3**^**]**	**Tumour volume [mm**^**3**^**]**	**Tumour volume [mm**^**3**^**]**	**Tumour volume [mm**^**3**^**]**	**Tumour volume [mm**^**3**^**]**	**Tumour volume [mm**^**3**^**]**	**Tumour volume [mm**^**3**^**]**	**Tumour volume [mm**^**3**^**]**
**5**		7.26	13.42						
(♂, intact)									
**6**		0.531	11.78			699.19			
(♂, intact)									
**7**				1.37	1.51		2.14	11.43	36.50
(♂, intact)									
**8**	1.65	11.27	24.72			1,928.83			
(♂, castrated**)**									
**9**		8.66	32.42			1,533.26			
(♂, castrated**)**									

The male animals received 10^3^ MnCl_2_ labelled cells. The tumour volumes are displayed for the respective days of MRI measurements after subcutaneous injection.

♂ = male animal.

**Table 5:** **Analysis of tumour volumes detected via 7 T** ***in vivo*** **MRI in a CT1258 based** ***in vivo*** **model of prostate cancer**

**Animal**	**Day 23;**	**Day 25;**	**Day 39;**	**Day 47;**
	**Tumour volume [mm**^**3**^**]**	**Tumour volume [mm**^**3**^**]**	**Tumour volume [mm**^**3**^**]**	**Tumour volume [mm**^**3**^**]**
**10**	5.16	10.76	420.45	
(♀, intact)				
**11**		1.33	1.18	3.60
(♀, intact)				
**12**	8.73	12.3	271.19	
(♀, OHM)				
**13**	19.48	29.75	148.65	
(♀, OHM)				

The female animals received 10^3^ MnCl_2_ CT1258 labelled cells. The tumour volumes are displayed for the respective days of MRI measurements after subcutaneous injection.

♀ = female animal; OHM = Ovariohysterectomised.

### Autopsies and histological analyses

All tumours were located subcutaneously, were non-adhesive to the surrounding tissue and its diameters varied from 10 to 15 mm. Some tumours developed ulceration while animals showed no signs of metastasis or of invasive tumour growth. Histological analysis of the tumours showed pleomorphic cells with a high mitotic index (Figure 5 C and D). The centres of the obtained tumours were highly necrotic (Figure 5 C). Most of the tumours infiltrated into the subcutaneous fat tissue (Figure 5 D) and some tumours developed an incomplete fibrous capsule.

## Discussion

One of the major challenges of this study was the identification of adequate MnCl_2_*in vitro* cell labelling parameters due to the cellular toxicity of MnCl_2_[27]. We aimed at identifying dosages as low as possible but still high enough to produce a robust MRI contrast. Incubation of approximately 1x10^7^ cells with 0.035 M MnCl_2_ for 1.5 h was evaluated to be suitable for cell labelling as comparable effects on viability and cell proliferation were observed. The labelling reaction resulted in cell viability of 89–90% and *t*-test analysis of cell proliferation resulted in a p value of p = 0,008 matching the viability test results. After the labelling reaction, the medium was discarded, and the cells were further cultivated in non-labelled culture medium overnight. This ensured the recovery of cells and removal of residual MnCl_2_. Prior to *in vivo* injections, the labelled cells were washed again to ensure that the positive signal enhancement in T1 weighted MRI is generated by the labelled cells and not residual MnCl_2_ in the injected solution.

The labelling parameters reported for human lymphocytes, embryonic stem cells and bone marrow stromal cells differed from the ones of the presented study. Concentrations of labelling MnCl_2_ solutions reported in the literature range from 0.1 to 0.5 mM and therefore, are lower than the concentration used in our study.

The therein reported incubation time spans did not exceed 30 to 60 min and - apart from the lymphocyte study with a cell number of approximately 2.4x10^7^ - the applied absolute cell numbers were lower (3x10^6^) than the cell number applied in the present study [23,31-33]. The reported labelling reactions were uniformly performed in 0.9% sodium chloride solutions, whereas the labelling reactions within the present study were performed directly in culture medium. This might have led to higher tolerated concentrations of labelling MnCl_2_ showing no major cytotoxic effects. During our labelling reactions, the respective cells remained in a buffered medium system and were supplied with nutrients in order to ensure their viability.

After labelling of the canine CT1258 cells with MnCl_2_ and SPIOs, an *in vitro* 1 T MRI pre-study using an agar phantom was performed. In general, agar phantoms share similar MRI characteristics with tissue during MRI. This part of the study was performed to evaluate whether labelled cells are also detectable at low magnetic fields (absolute cell number detection limit) as in most cases of human and veterinary examinations, MRI devices with magnetic field strengths of one to three Tesla are used. Thus, besides the herein generated data for basic research a potential translation into clinical setups was evaluated. Furthermore, the *in vitro* pre-study allowed a reduction of animal numbers for subsequent *in vivo* studies.

MnCl_2_ and SPIOs labelled CT1258 cells showed the same *in vitro* detection limit accounting 1x10^5^ cells. In the literature, lower numbers of SPIO labelled cells have been detected *in vitro* (as reviewed for example in [20,37]) while detection of labelled MnCl_2_ cells was reported for cell numbers exceeding 1x10^6^ using MRI systems with a magnetic field strength of more than 1 T [23,31-34,36]. However, it should be considered that within these studies, determination of manganese concentrations or characterisation of relaxation properties represented the primary study goals. In contrast, the evaluation of cell detection thresholds was not a primary objective of these studies. Nevertheless, the results of our study demonstrate that cell numbers lower than those already reported are sufficient for *in vitro* MRI detection.

Within the MRI scans of the first animal group the MnCl_2_ labelled cells were detectable for a longer time span than SPIOs labelled cells. The MnCl_2_ labelled cells were locatable until nine days after injection in T1 weighted MRI scans. The cells were not detectable in T2 and T2* weighted MRI scans and this makes false positive interpretation about the position of the cells unlikely. SPIOs labelled cells were detected up to four days after injection in T2 and T2* weighted MRI. On the following days, an exact distinction of cells and surrounding tissue was not possible. Generally, SPIOs generate a loss of signal on MRI and depending on the localisation, it is difficult, to distinguish the signal voids caused by SPIOs from other sources of hypointense MRI signals such as motion artefacts, susceptibility artefacts, bleeding/hemosiderin, calcification, water-fat interfaces, and air [23,35,36].

Thus, we decided to use only MnCl_2_ for the subsequent *in vivo* MRI scans in the second group of animals. Moreover, as manganese is transported actively through Ca^2+^ channels into biologically active cells, MRI enables a correlation between cellular viability and a T1-weighted positive signal [23]. Thus, information about the localisation and survival of administrated cells like in injured myocardium was generated [31-33]. SPIO labelling in contrast, does not provide any biologic information of the labelled cells, as residual SPIOs particles from dead SPIO-labelled cells are also phagocytised non-specifically by macrophages from the surrounding tissue [23,33]. After death of manganese labelled cells, Mn^2+^ diffuses passively out of these cells resulting in reduced T1-shortening effect and loss of contrast effect [23,36]. This was also observed during the present study as the signal intensity of MnCl_2_ labelled cells decreased (within the first group (n = 2) until 9 days after injection, within the second group (n = 18) until 16 days after injection) in a time dependent manner. In an *in vivo* study by Chung et al. [23], MnCl_2_ labelled human embryonic stem cells (hESC) were detected through 3 T MRI until four to five days after injection. Our scans were performed in a 7 T system which might have led to the detection during longer time periods. However, in most cases, the MnCl_2_ labelled cells were detected during the first five days comparable to the previously reported findings [23]. Regarding the cell numbers injected (10^3^ and 10^4^), no significant correlation between cell numbers and signal intensity was observed. Within a total of 18 animals which were part of the second group of animals, 13 animals developed tumours which mimicked the natural behaviour of the original tumour. This demonstrates that MnCl_2_ labelling did not alter the biological activity and characteristics of CT1258 cells.

In conventional *in vivo* studies characterising tumour development in rodent models as the study by Fork et al., the tumour development was analysed via manual palpation whereby the tumours were allowed to grow to a size ranging from 5 to 8 mm which lasted 20 to 42 days after s.c. injection of CT1258 cells [14]. In the present study, the two animals inoculated with unlabeled cells showed comparable results to the Fork study. Due to the use of contrast enhanced 7 T MRI scans, the induced tumours were detected prior to manual palpation and in some cases earlier than 20 days after injection although lower cell numbers were applied. Taken together, the time spans to sacrification of the unlabelled animals and the MRI contrast group were comparable to those of the Fork study.

Furthermore, the comparison of the tumours in the male animals showed a correlation between cell number injected and the tumour size and disease progression. Animals of both genders which had received 10^3^ cells, developed tumours after comparable time periods ranging from 17 to 25 days following injections. We did not observe significant gender-dependent and hormone-status dependent effects on tumour size and progression.

Consistent with observations by Fork et al. [14] no animal developed metastases which might have been caused by fast tumour progression leading to euthanasia of the animal. Moreover, there seems to be a low disposition of CT1258 cells to metastasise as the primary tumour was obtained from a 10 year old dog which -despite of several small metastases in the mesentery – showed no signs of abnormalities [13].

The histological analysis of the tumour masses showed cells with characteristic appearances and morphologies of a prostate adenocarcinoma, comparable to the findings which were reported during establishment of the cell line [13,14].

## Conclusions

In conclusion, we demonstrate that labelling cells of the prostate cancer cell line CT1258 with MnCl_2_ and SPIOs represents a feasible method allowing detection through MRI in *in vitro* and *in vivo* models. This is the first study reporting MnCl_2_ labelling and *in vivo* tracing of prostate cancer cells allowing monitoring of tumour development in early stages *in vivo* prior to manual palpation. The opportunity to characterise early tumour development and cellular behaviour may be of major interest since it allows evaluation of agents intervening in early stages of *in vivo* tumour development. Thereby, the contrast enhanced MRI based visualisation allows calculation of actual tumour volumes at different points of time without scarifying the animal (Figure 5). Thus, in addition to a possible significant reduction of animals required for studies, the tumour development can be monitored and described in single individuals reducing inter-individual variance. The MnCl_2_ labelled prostate cancer cells kept their highly tumourigenic potential in male and female NOD-scid mice indicating that the labelling does not majorly affect the cellular behaviour. Furthermore, histological analyses of the induced tumours showed the same characteristics as described for the original tumour. Consequently, the possible contrast enhanced *in vivo* visualisation of early tumour stages may be of significant benefit for the evaluation of therapeutic strategies in *in vivo* animal models used in veterinary and human medicine.

## Methods

### Cell line

The characteristics, cultivation conditions, and a basic *in vivo* growth pattern were previously described for the canine prostate cell line CT1258 [13,14].

### *In vitro* labelling of CT1258 cells with MnCl_2_

MnCl_2_·4H_2_O (Appli-Chem, Darmstadt, Germany) was dissolved to a concentration of 1 M MnCl_2_ and sterile-filtered. Aliquots were pipetted into CT1258 cell culture flasks and mixed with the culture medium (Medium 199 (Gibco, Karlsruhe, Germany) 20% heat-inactivated fetal calf serum (PAA Laboratories GmbH, Coelbe, Germany), 200 U/ml penicillin and 200 ng/ml streptomycin (Biochrom AG, Berlin, Germany)) to a final volume of 5 ml. Labelling of the cells was performed with the following MnCl_2_ concentrations: 0.02 M, 0.025 M, 0.03 M, 0.035 M, 0.04 M, 0.045 M. Incubation was performed for 1.5 h at 37°C and 5% CO_2_. Cells which were labelled with 0.035 M MnCl_2_ were also incubated for 3 h, 4 h, 5 h, 6 h and 24 h at 37°C and 5% CO_2_. After incubation the labelling medium was discarded, the cell layer washed with PBS and the cells further cultivated overnight in culture medium at 37°C and 5% CO_2_.

### *In vitro* labelling of CT1258 cells with SPIOs

According to an established protocol, 5x10^6^ cells were transferred in a culture flask with 5 ml culture medium and 41.15 μl Endorem infusion suspension (Guerbet S.A., Roissy, France) according to 130 pg iron oxide nanoparticles per cell. The cells were incubated overnight at 37°C and 5% CO_2_.

### Cell viability

Cell staining was performed with 500 μl 0.5% solution of Trypane-blue (Sigma Aldrich, Munich, Germany). The cells were incubated for 10 min at room temperature, the Trypane-blue solution was discarded, the cell layer washed with PBS, the cells trypsinised with 1 ml TrypLE Express (Invitrogen, Karlsruhe, Germany) and the cell number was determined.

### Cell proliferation assay

5x10^4^ MnCl_2_ labelled CT1258 cells/well were transferred into a 96 well plate (BD Falcon, Heidelberg, Germany) and incubated for 24 h in 100 μl culture medium at 37°C and 5% CO_2_. As control, unlabelled cells were co-incubated. Cell proliferation was evaluated using the Cell Proliferation ELISA, BrdU (colorimetric) kit (Roche Diagnostics, Mannheim, Germany) according to the manufacturer’s instructions. The measurements and data analyses were performed with Synergy 2 multi-mode microplate reader and the Gen5^TM^ software (BioTek, Bad Friedrichshall, Germany). A *T*-test was performed for analysis of the cell proliferation experiments.

### Agar phantom construction

A 1% agar (Invitrogen, Karlsruhe, Germany) gel solution was filled half-full into a an pipette tip box (Greiner Bio-One, Frickenhausen, Germany) and an unskirted 96 well PCR plate (Eppendorf, Hamburg, Germany) was embedded onto the liquid agar solution, so that sample wells were formed. The labelled cells were trypsinised, the cell number was determined, aliquoted into 1.5 ml reaction tubes (Eppendorf, Hamburg, Germany) and pelleted. The following cell numbers of either MnCl_2_ or SPIOs labelled cells were used: 7x10^5^, 2.5x10^5^, 10^5^, 5x10^4^, 2.5x10^4^, 10^4^, 5x10^3^, and 10^3^. As controls 1 μl 1 M MnCl_2_ solution, 1 μl Endorem solution, 10^5^ unlabelled cells and 30 μl culture medium were used. The cell aliquots and controls were mixed with 30 μl hand-warm 4% gelatine solution and the samples were pipetted into the wells of the polymerised agar phantom. The air bubbles were removed. After polymerisation, the phantom was covered with 1% agar gel and air bubbles were removed. The plastic box was removed prior to MRI scans.

### Animals

Prior to the MRI contrast scanning analysis two male NOD.CB17-*Prkdc*^*scid*^/J (in the following NOD-Scid) mice were inoculated with 10^4^ unlabelled CT1258 cells subcutaneously at the abdominal flank. We monitored the tumour growth by regular daily observation and manual palpation.

The MRI contrast study involved 20 NOD.CB17-*Prkdc*^*scid*^/J mice (6 female, 14 male). Three female and six male animals were ovariohysterectomised/and castrated, respectively.

All animals were bred and maintained in a protected environment at the Central Animal Facility of the Hannover Medical School. Mice were fed autoclaved food and water and any manipulation was performed in a laminar flow hood. The animals were observed daily and euthanisation was carried out if necessary, depending on the clinical condition, tumour size, and occurrence of tumor mass ulceration. The animals were euthanised by exsanguination after inhalation of >70% CO_2_. The study was approved by the Lower Saxony State Office of Consumer Security and Food Safety (33-42502-05/950).

### Inoculation of labelled cells

Aliquots of the respective cell number/animal (either 10^3^ or 10^4^ cells) were transferred into 1.5 ml cups. The cells were centrifuged for 10 min at 1,000 rpm and the cell pellet was washed twice with sterile PBS. The cells were resuspended in 200 μl PBS, aspirated into Insulin-Syringes (BD Micro-Fine, BD, Heidelberg, Germany), and injected subcutaneously into the abdominal flank of the respective animals.

Altogether 19 animals were inoculated with 10^3^ (6 female, 8 male) or 10^4^ (5 male) MnCl_2_ labelled cells. One male animal was inoculated with 10^4^ SPIOs labelled cells.

### Autopsy and histological staining

Animals were autopsied immediately after euthanasia. Tumour masses were fixed in 4% buffered formalin (pH 7.2) embedded in paraffin. Sections (4 μm thick) were stained with hematoxylin and eosin.

### MRI Scans

The agar phantom was scanned with a 1 T MRI system (Siemens Magnetom Expert, Erlangen, Germany) and data were analysed with dicomPACS version 5.2 (Oehm and Rehbein, Rostock, Germany). T1 weighted MRI scanning parameters were as follows: pulse repetition time (TR) = 330 ms, echo time (TE) = 12 ms, flip angle (FA) = 90°, slice thickness (ST) = 3 mm. T2* weighted MRI scanning parameters were: TR = 800 ms, TE = 26 ms, FA = 20°, ST = 2 mm.

*In vivo* MRI was performed on a 7 T Bruker Pharmascan 70/16 (Bruker Biospin, Ettlingen, Germany) under Paravision 5.0 equipped with a 6 cm Volume Resonator. Anaesthesia of the animals was maintained with a concentration of 1–2% isoflurane and the body temperature was held at approximately 37°C using a temperature control unit (Small Animal Instruments, Stony Brook, NY, USA). The animals were divided into two groups.

Within the first group, two males received 10^4^ cells labelled with either MnCl_2_ or SPIOs subcutaneously into the left abdominal flank. The animals were scanned on day 1, 4, 9, 15 and 28.

Although in general RARE and FSE MRI sequences are very similar we have chosen the standard Bruker Turbo-RARE for easy comparison with other groups and a modified FSE Protocol for the detection of our labeled tumour cells.

In order to nearly null the signal from the surrounding tissue an Inversion pulse before the normal pulse sequence is applied. The FSE tailored to T2 contrast has decreased scan time allowing for more signal averages and higher resolution. The drawbacks are some edge blurring and brighter fat due to lack of J-coupling. Scan parameters were as follows: Fast Spin Echo (FSE) T1: TR = 1300 ms, TE = 9.5 ms, ST = 1 mm; FSE T2: TR = 2500 ms, TE = 36 ms, 2 echoes; (Turbo-RARE) Rapid Acquisition with Relaxation Enhancement T2: TR = 2500 ms, TE = (eff) 36 ms, 1 echo, ST = 1 mm; T2*: Multi Gradient Echo (MGE) TR = 2000 ms, TE = 9 ms, FA = 30°, ST = 1 mm. The second group contained 18 animals (6 female, 12 male) which received either 10^3^ or 10^4^ MnCl_2_ labelled cells subcutaneously into the right abdominal flank. The animals were divided into two subgroups. In the first subgroup (n = 8; 4 castrated, 4 intact male mice) the animals were scanned on days 1, 4, 8, 10, 15, 17, 22, 24, and 43. The animals of the second subgroup (n = 10; 3 ovariohysterecotmised, 3 intact female mice, 2 castrated, 2 intact male mice) were scanned on days 2, 10, 16, 23, 25, 39, 47, 53, 60, and 67. Scan parameters were as follows: RARE T1: TR = 1300 ms, TE = 9 ms, ST = 1 mm, Turbo-RARE T2: TR = 2500 ms, TE = (eff) 36 ms, 1 echo, ST = 1 mm.

### MRI data analysis

MRI image data were analysed with the ITK-SNAP 2.1.4-rc1 software [38] (http://www.itksnap.org/). Tumour sizes in [mm^3^ were determined threefold and the mean value was calculated.

## Competing interests

The authors declare that they have no competing interests.

## Authors’ contributions

KAS cell culture work, labelling with MnCl_2_, viability and cytotoxicity testing, assistance with agar phantom construction, assistance with MRI scans and interpretation of data, analysis of tumour volumes, partial manuscript drafting, MM performed MRI scans and supervised the data analyses, partial manuscript drafting and study design, SG performed castration of mice, autopsy and immunohistochemistry, partial manuscript drafting, MM2 performed the *in vivo* inoculation of the NOD-Scid mice, SW performed the ironoxide nanoparticle labelling and agar phantom construction, PW performed the *in vivo* inoculation of the NOD-Scid mice, MD supervised the *in vivo* work, partial study design, partial manuscript drafting, JB, head of the Centre for Human Genetics Bremen, performed partial study design, HME partial study design, coordination and supervision of cell culture and contrast agent labelling and *in vitro* work, partial manuscript drafting and finalisation, HJH, head of the Institute of Laboratory Animal Science, performed partial study design, approved the final manuscript, IN incited the study and coordinated the operational procedure, approved the final manuscript. All authors read and approved the final manuscript.

## Pre-publication history

The pre-publication history for this paper can be accessed here:

http://www.biomedcentral.com/1471-2407/12/284/prepub
